# Social determinants across the MDR/RR-TB care cascade: a critical narrative review

**DOI:** 10.3389/fpubh.2026.1993300

**Published:** 2026-10-30

**Authors:** Maciej Burski, Katarzyna Tuzimek, Kateryna Szapowal, Maria Korzeniewska-Koseła, Adam Nowiński

**Affiliations:** Department of Tuberculosis Epidemiology and Surveillance, Institute of Tuberculosis and Lung Diseases, Warsaw, Poland

**Keywords:** MDR/RR-TB, public health, social determinants, social factors, treatment outcomes

## Abstract

**Introduction and aim:**

Multidrug- and rifampicin-resistant tuberculosis (MDR/RR-TB) remains a major public health challenge, particularly in the WHO European Region. This critical narrative review examines how social determinants influence the clinical course and outcome of patients across the MDR/RR-TB care cascade, from access to diagnosis and treatment initiation to treatment support, retention in care.

**Methodology/Scope:**

Researchers reviewed the current publications on the topic of our review until June 2026. Sources were prioritized on the basis of relevance to MDR/RR-TB, social determinants, and care-cascade outcomes. Evidence from broader TB populations was used only when MDR/RR-TB-specific data were limited and is identified as such.

**Main findings:**

Across settings, poverty, catastrophic total costs, homelessness, incarceration, stigma, education, mental-health difficulties, substance use, and barriers related to legal status, mobility, language, insurance, and access to care were associated with delayed diagnosis. Material, psychosocial, and individualized treatment support may improve retention and outcomes, but much of the evidence is observational and context-specific.

**Conclusion:**

Social determinants should be addressed throughout the MDR/RR-TB care cascade through patient-centered models that combine effective regimens with financial, psychosocial, housing, and individualized adherence support. The evidence base remains heterogeneous, and causal claims should be interpreted cautiously.

## Introduction

Despite more effective prevention and treatment options, tuberculosis remains one of the most serious threats to public health and is among the leading causes of death from infectious diseases worldwide. Most difficult to control is tuberculosis caused by drug-resistant *Mycobacterium tuberculosis* strains. Throughout this review, MDR/RR-TB denotes rifampicin-resistant TB, including multidrug-resistant TB, which is resistant to at least rifampicin and isoniazid. According to estimates, there are more than 10 million new cases of tuberculosis annually worldwide, including nearly 400,000 cases of MDR-TB. The mortality rate remains at 1.23 million deaths per year (1). The World Health Organization (WHO) European Region, which includes Central and Eastern Europe and Central Asia, accounts for one-fifth of all MDR-TB cases worldwide, despite representing only about 2–3% of the total number of tuberculosis cases (2). In Poland, 3,965 cases of tuberculosis were reported in 2024, including 106 cases of MDR-TB (3).

In the modern approach to tuberculosis treatment, it is increasingly recognized that the causes of treatment failure in MDR-TB extend beyond biomedical factors, including the efficacy of the therapies used (4). The recovery process for a patient with multidrug-resistant tuberculosis involves a prolonged, often toxic treatment lasting up to several months, which affects not only the body but also emotional and financial aspects as well as interpersonal relationships (5). Low economic and social status, housing issues, or limited access to healthcare services can pose a barrier to treatment adherence. Discontinuation of therapy due to non-medical factors poses a threat not only to the patient’s life and health but also to those around them, creating an environment conducive to the spread of MDR-TB, thereby translating social factors into a public health threat (6).

## Objectives of the study

The aim of this article is to critically review how social determinants influence the MDR/RR-TB care cascade, including exposure and transmission, access to diagnosis, treatment initiation, retention in care, adherence, treatment outcomes, and acquisition or amplification of additional drug resistance. Particular attention is given to poverty and income, housing instability and homelessness, migration-related barriers, incarceration, stigma, education, mental health, and substance use.

Given that multidrug-resistant tuberculosis poses a major challenge in the Central and Eastern Europe and Central Asia regions, understanding these mechanisms is crucial to controlling the MDR-TB epidemic.

## Review methodology

This article was developed as a critical narrative review.

A semantic search was conducted using PubMed and Google Scholar with relevant keywords as above. Additionally, for each key article, the reference list was analyzed to identify earlier studies on which it was based. More recent articles citing the key publications were also identified. Peer-reviewed studies and authoritative institutional reports were considered when they addressed a social determinant and at least one point in the TB care cascade. MDR/RR-TB evidence was prioritized; general TB evidence was included only where MDR/RR-TB-specific evidence was limited. No formal risk-of-bias tool or quantitative pooling was performed, which is an acknowledged limitation of this critical narrative review. Selection was purposive rather than exhaustive; evidence from Africa, Asia, Latin America, and Europe was synthesized thematically. In Table 1, DR-TB includes MDR/RR-TB studies, while TB denotes populations not selected for drug resistance.

**Table 1:** Characteristics, selected findings, and principal limitations of key studies discussed in the review.

Setting	Ref.	Design and sample	Population, outcome / Finding	Principal limitation
Eritrea	(5)	Qualitative study; 12 patient interviews, 3 focus groups (*N* = 24), and 3 staff interviews (total *N* = 39)	Pulmonary TB under DOTS; adherence barriers, knowledge gaps, food insecurity, side effects, and family support	Context-specific qualitative design in urban Asmara; non-generalizable to rural Eritrea
Eastern Cape, South Africa.	(6)	Qualitative explorative study; professional nurses from six rural clinics	Rural TB care delivery; multilevel drivers of treatment interruption, transport, and food insecurity	Provider perspective only; limited to six rural clinics in one subdistrict
Kenya	(7)	Mixed methods; 217 participants; completion data for 165 patients	DR-TB; psychosocial support, depression, completion: 73% (*n* = 81) vs. 56% (*n* = 43)	Cross-sectional and non-randomized; urban program data
Bangladesh	(8)	Conceptual narrative review; synthesis of national surveys and empirical studies	Vulnerable populations in Bangladesh; poverty, hidden out-of-pocket costs, and care fragmentation	Secondary literature review; no primary patient data collected
HIC. MIC and LIC	(9)	Systematic review and meta-analysis; 11 eligible studies encompassing *N* = 817 MDR-TB patients/households	MDR-TB patients and households; catastrophic costs, direct vs. indirect medical expenses, and financial coping mechanisms	Scarce evidence in high-burden countries; high heterogeneity and potential publication bias
São Paulo, Brazil.	(10)	Qualitative study; in-depth interviews with *N* = 7 MDR-TB patients	Urban MDR-TB; COVID-19 care barriers, economic distress, side effects, and health system access	Small sample size (*N* = 7); single center during pandemic context
Guizhou, China	(11)	Observational household study; *N* = 180	MDR-TB; CHE, catastrophic total costs (80.6% of households), mobility, LTFU	Single province; associations do not establish causality
Pakistan	(12)	Qualitative study; in-depth interviews with *N* = 39 DR-TB patients lost to follow-up	DR-TB LTFU; patient beliefs, drug side effects, economic burdens, and health system distance	Patient self-report bias; exclusion of non-reachable patients and provider perspectives
Pune, India.	(13)	Quantitative cross-sectional study; *N* = 64 DR-TB patients and caregivers	DR-TB; quality of life, physical/psychological challenges, environmental barriers	Small sample size; single-center hospital design and selection bias
Johannesburg, South Africa.	(14)	Cross-sectional survey; *N* = 239 adult TB patients	General TB (urban adults); unemployment 50.2%; syndemic clustering of HIV, alcohol misuse, smoking, and legal status	Cross-sectional design; single metropolitan area limiting generalizability
Haiti	(15)	Mixed-methods cohort and qualitative study; 17 MDR-TB patients and 16 caregivers	Rural MDR-TB; nutritional status, social support, diagnostic delays, and treatment response	Small sample size; limited geographic scope in two rural clinics
Anhui, China.	(16)	Population-based retrospective epidemiological study; *N* = 211,892 pulmonary TB cases	General pulmonary TB; epidemiological trends, demographic predictors, and spatial distribution	Passive surveillance system; potential underreporting and lack of individual clinical data
Hubei, China.	(17)	Cross-sectional comparative study; *N* = 292 MDR-TB patients (73 migrants, 219 residents)	Internal migrant MDR-TB; hukou status, insurance coverage, out-of-pocket costs, and non-adherence	Cross-sectional single-center study; inability to confirm causal direction
Peru	(18)	Nested case–control study; *N* = 2,337 TB cases and *N* = 981 community control households	General TB: urban households; multidimensional poverty, underweight, social capital, and PAFs	Observational design; potential reverse causality between TB and poverty/underweight
Catalonia, Spain.	(19)	Observational descriptive study; *N* = 121 migrant TB patients	General TB among migrants; language barriers in 66.1% of program referrals, diagnostic delay, community health worker interventions, and retention in care	Observational design without control group; missing survey responses
Brazil	(20)	Retrospective national cohort study; *N* = 284,874 TB cases (7,749 homeless, 277,125 housed)	General TB: homeless vs. housed; unsuccessful outcomes, substance use, psychiatric disorders, and DOT effect	Secondary surveillance data; missing variables and potential reporting bias
Brazil	(22)	Retrospective surveillance cohort; notified incarcerated TB cases (2008–2017)	General TB among incarcerated persons; unfavorable treatment outcomes, HIV/AIDS, comorbidities, and DOT coverage	Secondary surveillance data; incomplete records and unmeasured confounding
Latin America and the Caribbean.	(23)	Critical narrative review; synthesis of regional carceral health reports	Incarcerated populations; carceral overcrowding, intra-prison transmission, and continuity of care	Narrative review; substantial heterogeneity across regional carceral systems
Global policy analysis based on WHO/WISH 2024 framework.	(24)	Strategic policy analysis; synthesis of WHO/WISH report and 7 international case studies	Migrant and refugee populations; legal barriers, healthcare access, financing gaps, and policy frameworks	Policy analysis document; absence of primary empirical clinical patient data
England.	(25)	Mixed-methods study; retrospective cohort *N* = 2,252 TB patients and 25 qualitative interviews	General urban TB; vulnerability factors, migrant origin, housing precarity, and treatment outcome	Missing homelessness data in one site; small interview sample; excluded extrapulmonary TB
Johannesburg, South Africa	(26)	Qualitative; 16 people with DR-TB and 8 family members	DR-TB with prior disengagement; barriers and facilitators to retention	Purposive sample enriched for disengagement; limited generalizability
African countries	(27)	Scoping review; synthesis of 20 quantitative studies (*N* = 276,751 DR-TB patients)	African DR-TB; socioeconomic drivers, catastrophic out-of-pocket costs, job loss, and transport barriers	Restricted database search (2011–2020); limited number of quantitative studies
Global	(28)	Systematic review; 7 publications on TB in people experiencing homelessness	Homeless TB; unfavorable treatment outcomes, substance use, comorbidities, and DOT impact	Small number of eligible studies; high methodological heterogeneity
Puducherry, India	(29)	Retrospective outpatient cohort; *N* = 192	MDR/RR-TB; treatment outcome and clinical/social factors	Secondary records; residual confounding and missing variables
Poland	(30)	Retrospective national cohort study; *N* = 19,217 adult TB cases (2011–2016)	General TB in adults with comorbidities; mortality, treatment failure, HIV, alcoholism, and diabetes	Registry data capturing single main comorbidity; missing variable exclusions
Eight LMICs	(31)	Systematic review/meta-analysis; 10 studies, 1,621 patients	DR-TB; social-support packages, higher success (OR 2.58) and lower LTFU (OR 0.17)	No eligible RCTs; limited studies and substantial heterogeneity for LTFU
Southern Ethiopia.	(32)	Retrospective cohort study; *N* = 276 confirmed pulmonary MDR-TB patients	Pulmonary MDR-TB; treatment success rate, HIV co-infection, low BMI, and unevaluated cases	High proportion of unevaluated records (28.9%); single-center retrospective missing data
Five EECA countries	(33)	Multicountry observational analysis; *N* = 212	DR-TB; contributors to treatment outcome	Context-specific program data, largely preceding current short all-oral regimens
HIC. MIC and LIC	(34)	Scoping review;	Global MDR-TB; social determinants, multidimensional poverty, housing, and healthcare barriers	Scoping review methodology; lack of meta-analytic pooling or risk-of-bias scoring
Tamil Nadu, India.	(35)	Case–control study; *N* = 80 patients (20 MDR-TB cases, 60 DS-TB controls)	Confirmed TB; socioeconomic predictors of MDR-TB, household income, employment, and housing	Small sample size; retrospective exposure recall bias; single district
Poland	(36)	Descriptive case series / experience report; 39 complex TB cases (35 MDR-TB)	MDR-TB physicians; regimen optimization, drug resistance management, and drug access	Descriptive report; lacks systematic longitudinal patient outcome tracking

## Results

The following is a summary of the social determinants influencing treatment outcomes, treatment adherence, and mortality from MDR/RR-TB and, where indicated, broader TB populations, with a particular focus on poverty, income, homelessness, migration, incarceration, stigmatization, education, and substance use. The studies included in this review employed a variety of methodological approaches, including mixed-methods, cross-sectional, cohort, case–control studies, and systematic reviews, and their geographic scope spans low-, middle-, and high-income countries in Africa, Asia, Latin America, and Europe. Findings from general TB cohorts are presented as contextual evidence.

## Socioeconomic influence

The impact of socioeconomic factors on the course of MDR-TB treatment is multifaceted and represents one of the main barriers to global goals for the elimination of the disease (7, 8). Among these factors, three aspects can be distinguished: catastrophic costs and the financial burden on households, poverty and its impact on treatment adherence, and educational level and cognitive barriers.

The economic burden of MDR/RR-TB may be substantially greater than that of drug-susceptible TB (9). In one study, expenses related to treating a family member with MDR-TB may exceed 20% of annual household income, the WHO threshold for catastrophic total TB costs (10). Additionally, other MDR/RR-TB costs include direct medical costs such as medications expenditure, and hospitalizations; direct non-medical costs related to transportation to the hospital, lodging for the patient’s caregiver, and meals; and indirect costs associated with the inability of patients and their caregivers to work. In Guizhou, China in the 180 households studied, catastrophic health expenditure and TB-specific catastrophic total costs occurred in 51.7 and 80.6%, respectively (11). These estimates describe one provincial setting and should not be interpreted as universal rates.

Economic status and tuberculosis exhibit a bidirectional relationship—poverty increases the risk of contracting the disease, which in turn leads to a deterioration in economic status (11–13). MDR-TB also affects people of working age, leading to job loss (7, 12, 13). For example, in Johannesburg, the unemployment rate among general TB patients was 50.2% (14). Poverty and unemployment may delay the initiation of MDR-TB treatment and, by worsening treatment outcomes, may constitute a barrier to adherence and survival (10, 15). Financial burdens are associated with treatment discontinuation and, consequently, higher mortality. To mitigate this effect, it is necessary to implement economic support mechanisms (10).

A low level of education is associated with poorer treatment outcomes and may be partly related to health-literacy and adherence barriers (16, 17). People with low levels of education hold misconceptions about the causes of the disease and its transmission. Inadequate preventive health care contributes to the spread of tuberculosis and delays the decision to seek treatment (8, 14). In a general TB study in Peru, education was associated with lower TB burden (18).

## Vulnerable population impact

MDR-TB is not evenly distributed throughout society and primarily affects socially vulnerable populations. People with MDR-TB face biological, social, and systemic barriers that often compound and accumulate, leading to poorer treatment outcomes (8, 19).

Homelessness may contribute to treatment failure of tuberculosis sufferers. Prioritizing shelter and food over other needs health included, as well as the lack of a place to store medications, may contribute to poor treatment adherence (20, 21).

Correctional settings can intensify TB transmission through overcrowding, poor ventilation, delayed diagnosis, limited drug-susceptibility tests access. The inmate population exhibits a higher rate of adverse outcomes. The main factors include comorbidities (HIV infection, diabetes), self-medication, and social determinant of care during transfer or after release (22, 23). Evidence from Latin America and the Caribbean describes a high drug-resistant tuberculosis (DR-TB) burden and prison-associated transmission, but the available literature is heterogeneous and frequently focused on biomedical surveillance. WHO-recommended responses include systematic screening, rapid diagnosis and drug-susceptibility testing, effective treatment, infection prevention and control, and continuity of care. Remaining implementation gaps include linkage to community services after release, social protection, and rights-based conditions of detention (22, 23).

Migration is not a biological risk factor for MDR/RR-TB. Rather, legal status, mobility, language, health insurance, continuity of records, and access to affordable care can influence exposure, diagnostic delay, treatment initiation, and retention (19, 24, 25). In Catalonia, language and cultural barriers were reported in 66.1% referrals to a specialized support program for migrants; this is a program-specific figure rather than a general estimate for migrants with MDR/RR-TB (19). In a cross-sectional study of internal migrants with MDR-TB in Wuhan, non-adherence was associated with migration-related restrictions on insurance coverage and financial difficulties; the design does not establish migrant status itself as a causal risk (17).

The co-infection of HIV and MDR-TB is a classic example of the interaction between two diseases exacerbated by social factors (14). P HIV-positive patients with MDR-TB require closer monitoring, and lack of access to antiretroviral (ARV) therapy drastically worsens their prognosis (21).

## Psychosocial determinants

Psychosocial determinants, known as mediating factors, translate structural conditions into health behaviors and clinical outcomes (8, 9). Barriers to MDR-TB treatment create a network of connections between the environment and the patient’s mental state, which affects cooperation in the therapeutic process (26).

Stigmatization is one of the most significant barriers affecting the diagnosis and treatment of tuberculosis. Among general TB patients in Johannesburg, 29.3% experienced gossip about themselves, 24% were insulted or mocked, and 17.6% felt a sense of “impurity” after hearing their diagnosis (14). The fear of rejection by family and friends may delay treatment initiation and subsequent follow-up care (12, 19, 21). In some cases, fear of discrimination and stigmatization influences the decision to self-isolate, which results in the breakdown of family ties (27). In some communities, gender further influences stigmatization. In Pakistan, women often cannot decide on their own to seek treatment or visit a hospital without a male companion (12).

Psychological distress is much higher among patients with MDR-TB compared to those with drug-sensitive TB (26). 55% of DR-TB respondents in Kenya reported stress or depression during treatment (7). The main triggers are financial problems and unemployment, as well as having to take a large number of pills and the side effects of treatment (7). Mood disorders may contribute to a lack of motivation to continue therapy and treatment discontinuation (12).

Substance abuse, including alcohol, may contribute to unfavorable tuberculosis treatment outcomes, affecting patients both biologically and behaviorally (27, 28). Alcohol weakens the immune system and alters drug metabolism, potentially complicating treatment continuity, and contributes to increased hepatotoxicity and the occurrence of side effects. As a result, it increases the risk of treatment failure (29, 30). Among homeless individuals who inject psychoactive substances, the risk of treatment failure was 38–50% higher (20). Substance abuse is correlated with poverty, unemployment, and a lack of support, with these factors interacting with one another (27).

The important protective factor that mitigates the impact of economic determinants is social support (14, 19, 21). Support from a partner or someone involved in treatment provides emotional security and increases adherence (26, 31). In a Kenyan DR-TB study, support-group participation was associated with improved treatment adherence (7). Celebrating milestones in treatment or important life events together helps restore self-esteem (31). Higher social capital was associated with a lower risk of developing tuberculosis (18).

## Drug resistance interaction

The burden of drug-resistant TB reflects both transmission of already resistant strains and acquisition or amplification of resistance during treatment. Acquired resistance may occur when an effective multidrug regimen is not achieved because baseline or additional resistance is unrecognized, drug selection or dosing is inadequate, drug exposure is subtherapeutic, adverse effects are not managed, medicines are interrupted or unavailable, or diagnostic and health-system delays prevent timely regimen adjustment (10, 31–34). Social and structural barriers can contribute to these pathways but should not be framed as patient responsibility alone.

Treatment interruptions related to social barriers may contribute to resistance amplification alongside clinical and health-system factors (33). The clinical effectiveness of second-line drug therapy is linked to social support for patients. Combating drug resistance requires not only the use of appropriate antibiotics but also support for patients in overcoming the barriers that prevent the proper implementation of treatment regimens (34).

## Intervention effectiveness

Contemporary WHO guidance emphasizes patient-centered care rather than traditional facility-based directly observed therapy as the only model of treatment administration. Support may include self-administered treatment with individualized follow-up, community- or home-based treatment support, video-supported treatment, digital medication monitors, SMS or telephone contact, education, counseling, and material or psychological support. The modality should be selected according to the patient’s needs, preferences, clinical condition, available resources, and local context (21).

A 2020 systematic review of 10 studies involving 1,621 people with DR-TB, of which seven were eligible for meta-analysis, found that social-support packages containing material support were associated with higher treatment success (OR 2.58, 95% CI 1.80–3.69). Material support was also associated with lower loss to follow-up (OR 0.17, 95% CI 0.05–0.55), although heterogeneity was substantial for this outcome (I^2^ = 80%) (31). Because the pooled estimates rest on a small number of studies, they should be interpreted with caution and not translated directly into causal increases in individual probability. In the 2023 Kenyan mixed-methods study, treatment completion was reported for 73% of support-group participants (n = 81) and 56% of those not enrolled (n = 43), among 165 patients with available completion data (7). Because allocation to support groups was not randomized and the analysis was cross-sectional, this difference describes an association in that program rather than a causal effect of group participation.

Digital tools can support continuity of care for mobile populations through text messages, telephone contact, medication monitors, and video-supported treatment (19, 21).

Tuberculosis control programs tailored to local needs and integrated with the social welfare system yield the highest effectiveness (19). Shifting treatment to an outpatient setting with the option of home-based care eliminates geographic and transportation barriers, particularly among marginalized groups (24). The effectiveness of interventions for MDR-TB depends on a holistic support package provided in parallel with the treatment process (31).

## Discussion

The findings of this review suggest that MDR/RR-TB cannot be viewed solely as a biomedical phenomenon. It is, in fact, a “social disease,” whose course and treatment outcomes are influenced by the conditions in which people are born, live, and work (7, 13, 17). The collected data suggest that structural and psychosocial barriers, alongside biomedical challenges, remain important obstacles to achieving the goals of the “End TB” strategy (6, 21, 34).

### Poverty and the “catastrophic costs” mechanism

The high economic burden on patients with MDR-TB observed in the results (often greater than for drug-susceptible TB) may help explain associations between poverty and the risk of treatment failure (8). This mechanism is based on so-called catastrophic costs, which affected 80.6% of MDR-TB households in one provincial study (11). Poverty may force patients to make tragic choices between buying food and continuing treatment, leading to treatment discontinuation (loss to follow-up) (23, 24). A lack of financial security, exacerbated by job loss due to illness (often among the primary breadwinners), may contribute to poorer adherence, which in turn is a possible contributor to the acquisition of additional mycobacterial resistance (10, 14, 24, 28).

Much of the evidence synthesized here was generated when MDR/RR-TB treatment commonly lasted 18–24 months and frequently included injectable agents, high pill burdens, prolonged hospitalization, and substantial toxicity. WHO now prioritizes shorter all-oral regimens, including 6-month BPaLM/BPaL-based regimens for eligible patients and 9-month all-oral regimens in selected patients (1, 21). These regimens may reduce several adherence burdens, but they do not remove the effects of poverty, housing instability, stigma, mental-health needs, or disrupted access to care. Evidence from older regimens should therefore be interpreted separately from evidence generated under contemporary treatment.

### Housing and the environment as factors in transmission

An analysis of housing conditions helps us understand the dynamics of MDR-TB transmission. Overcrowding, poor ventilation, and homelessness are not merely correlates of the disease, but mechanical facilitators of the spread of mycobacteria (7, 13, 35). In settings such as slums, prisons, or homeless shelters, the high concentration of immunocompromised individuals (due to malnutrition or HIV coinfection) creates ideal conditions for epidemic outbreaks (19, 25). Homelessness, identified as a key factor in treatment failure, not only prevents the safe storage of medications but also makes it impossible to prioritize health over the need to find shelter, complicating outpatient treatment without appropriate support (19, 20).

### Barriers to access to care and diagnostic delays

Delays in initiating treatment, as observed in the analyzed studies, can be explained through the lens of systemic and geographic barriers. Long distances to medical facilities and high transportation costs can be substantial barriers for people in the lowest socioeconomic classes (5, 8, 22, 23). For migrants, these barriers are further exacerbated by their legal status and lack of health insurance, which may discourage some from seeking medical care out of fear of deportation or high costs (13, 16, 18, 21). Such delays not only worsen the patient’s prognosis but also prolong the period during which they remain infectious to others, potentially contributing to the transmission of MDR-TB in the general population (5, 18, 26).

### Stigmatization and mental health as mediating factors

Findings indicating a high prevalence of depression (over 50% of patients in some cohorts) and stigmatization shed light on the psychosocial burden of MDR-TB (6, 23). Stigma may lead to social isolation and the concealment of symptoms, which is a potential contributor to diagnostic delays and treatment discontinuation (5, 24). Fear of rejection by family or employers causes people to avoid seeking treatment at tuberculosis treatment facilities (5, 13).

The certainty of the available evidence is uneven. Qualitative studies provide detailed accounts of barriers but do not estimate their frequency; cross-sectional studies cannot determine temporal direction; retrospective cohorts are vulnerable to incomplete records and residual confounding; and several reviews combine MDR/RR-TB with broader DR-TB or general TB populations. Evidence on age and gender remains limited.

### Implications for Poland and central and Eastern Europe

Poland has historically relied heavily on hospital-based MDR/RR-TB treatment. Since 2022, the increase in notified MDR/RR-TB cases has been associated with a growing number of cases among people displaced from Ukraine and other foreign-born patients (35). This situation should be interpreted through continuity-of-care, legal, language, insurance, housing, and social-support needs rather than through nationality or migration as biological risk. Prolonged hospitalization solely to secure adherence, nutrition, or social support is difficult to justify when outpatient care is clinically appropriate.

A patient-centered model for Poland and neighboring countries should combine contemporary all-oral regimens with rapid drug-susceptibility testing, outpatient specialist care, individualized treatment support, material and psychosocial assistance, and cross-border continuity of records. Video-supported treatment can be offered as one flexible modality, but its effectiveness and cost-effectiveness among people displaced from Ukraine in Poland require formal evaluation rather than assumption (21, 36).

## Conclusion

Social determinants influence multiple points in the MDR/RR-TB care cascade, but their effects vary across populations, health systems, and treatment eras. Effective control therefore requires patient-centered clinical care combined with financial protection, stable access to medicines and diagnostics, housing and material support, mental-health care, and flexible adherence support. Future research should distinguish MDR/RR-TB from general TB populations, report setting and study design clearly, and evaluate social interventions alongside contemporary short all-oral regimens.
